# The regulatory effect of acetylation of HMGN2 and H3K27 on pyocyanin‐induced autophagy in macrophages by affecting *Ulk1* transcription

**DOI:** 10.1111/jcmm.16788

**Published:** 2021-07-18

**Authors:** Yu Du, Hongjun Guo, Lijuan Guo, Junming Miao, Hongyu Ren, Keyun Liu, Laibin Ren, Jinchen He, Xiaoying Wang, Junli Chen, Jingyu Li, Yi Wang, Ji Wang, Ning Huang

**Affiliations:** ^1^ Department of Pathophysiology West China School of Basic Medical Sciences & Forensic Medicine Sichuan University Chengdu China; ^2^ Department of Anesthesiology Nanchong Central Hospital Second Clinical Medical Institution North Sichuan Medical College Nanchong China; ^3^ Department of Obstetrics and Gynecology The First Affiliated Hospital of Zhengzhou University Zhengzhou China; ^4^ Department of Physiology School of Medicine Hubei University for Nationalities Enshi China; ^5^ Department of Anesthesiology Affiliated Hospital of North Sichuan Medical College Nanchong China

**Keywords:** acetylation, autophagy, H3K27, HMGN2, macrophages, pyocyanin

## Abstract

Pyocyanin (PYO) is a major virulence factor secreted by *Pseudomonas aeruginosa*, and autophagy is a crucial homeostatic mechanism for the interaction between the pathogens and the host. It remains unknown whether PYO leads to autophagy in macrophages by regulating histone acetylation. The high mobility group nucleosomal binding domain 2 (HMGN2) has been reported to regulate the PYO‐induced autophagy and oxidative stress in the epithelial cells; however, the underlying molecular mechanism has not been fully elucidated. In this study, PYO was found to induce autophagy in macrophages, and the mechanism might be correlated with the up‐regulation of HMGN2 acetylation (HMGN2ac) and the down‐regulation of H3K27 acetylation (H3K27ac) by modulation of the activities of acetyltransferases and deacetylases. Moreover, we further demonstrated that the up‐regulated HMGN2ac enhances its recruitment to the *Ulk1* promoter, while the down‐regulation of H3K27ac reduces its recruitment to the *Ulk1* promoter, thereby promoting or inhibiting the transcription of *Ulk1*. In conclusion, HMGN2ac and H3K27ac play regulatory roles in the PYO‐induced autophagy in macrophages.

## 1 INTRODUCTION

*Pseudomonas aeruginosa* (PA), a Gram‐negative pathogenic bacterium of the genus *Pseudomonas*, accounts for a wide variety of acute and chronic infections. Among the other systems in the body, the respiratory system is most susceptible to PA infection.^1^ PA can also infect the urinary system, blood system and central nervous system.^2^ Pyocyanin (PYO) is a major virulence factor secreted by PA.^3^ PYO can impose oxidative stress on the host cells and cause inflammatory damage by up‐regulating the pro‐inflammatory factors such as TNF‐α and IL‐8.^4^

Autophagy is a crucial homeostasis mechanism involved in many physiological and pathological processes.^5^, ^6^ PA has been well documented to induce autophagy in macrophages, mast cells and bronchial epithelial cells.^7^, ^8^, ^9^ Current studies on autophagy pertaining to PYO infection have been restricted to the astrocytes and bronchial epithelium.^10^, ^11^ Therefore, whether PYO can lead to autophagy in macrophages remains to be clarified.

Manipulating the host cellular epigenetics is one of the notable strategies by which bacteria evade immune responses. And it has been verified that H4K16ac plays an important role in the process of autophagy.^12^ Subsequent studies have indicated that H2AK5ac, H2BK15ac, H3K9ac, H3K56ac and H4K5ac also regulated autophagy.^13^, ^14^, ^15^ However, whether PA or PYO regulates autophagy via epigenetic manipulation has not yet been elucidated.

The high mobility group nucleosomal binding proteins (HMGNs) are the only non‐histone nuclear proteins capable of binding to nucleosomes, where HMGN2 is a critical member.^16^ We have previously reported that HMGN2 has antibacterial and antiviral effect.^17^, ^18^ Moreover, our previous studies have also found that HMGN2 can mediate antimicrobial peptide expression,^19^ induce cytoskeleton rearrangement^20^ and bacterial internalization,^21^ and modulate oxidative stress^22^ and autophagy,^23^ all of which contribute to innate immune responses.

This study aimed to investigate whether PYO induces autophagy in macrophages, to detect the changes in the acetylation of HMGN2 and H3K27 in RAW 264.7 cells both with and without *Hmgn2* knockout under PYO treatment and to explore the interactions between HMGN2ac and H3K27ac. We further explored the autophagy‐related genes that are regulated by modification in the acetylation of HMGN2 and H3K27. Since PA/PYO nosocomial infection is a challenging problem in clinical medicine, this study would be of great clinical significance.

## 2 MATERIALS AND METHODS

### 2.1 *Antibodies* *and reagents*

The rabbit polyclonal antibodies against HMGN2 (9437S), LC3B (3868S), p62 (8025S), acetylated lysine (9441S), H3 (4499S) and H3K27ac (8173T) were purchased from Cell Signaling Technology; the rabbit polyclonal antibody against p‐ULK1 (ab203207) was obtained from Abcam, while the rabbit anti‐β‐actin (AB0035) monoclonal antibody was from Abways, China, and the rabbit polyclonal antibody against ULK1 (A5149) was purchased from Bimake Corporation. The FITC‐conjugated goat anti‐rabbit IgG (A0562) was purchased from the Beyotime Institute of Biotechnology. Alexa Fluor 488–labelled goat anti‐rabbit IgG (GB25303) was purchased from Servicebio. The All‐in‐One cDNA Synthesis SuperMix was obtained from Bimake Corporation. The 2xSYBR Green RT‐qPCR Master Mix was procured from Vazyme. Chloroquine (CQ), trichostatin A (TSA), C646 and 3‐Methyladenine (3‐MA) were purchased from MedChemExpress. The RPMI 1640 medium was purchased from Gibco. PYO, N‐acetylcysteine (NAC), dimethyl sulphoxide (DMSO), 4′,6‐diamidino‐2‐phenylindole (DAPI) and H_2_O_2_ were purchased from Sigma. The foetal calf serum (FBS) was purchased from PAN‐Biotech (Germany), and the penicillin and streptomycin were obtained from Solarbio (Beijing, China).

### 2.2 *Cell* *culture*

The murine macrophage‐like cell line RAW 264.7 and human monocytic cell line THP‐1 were obtained from the Institute of Cell Biology, Chinese Academy of Sciences. The *Hmgn2*‐knockout (KO) RAW 264.7 cells were prepared using the CRISPR/Cas9 technique in our laboratory.^24^ The suspended THP‐1 cells were treated with 100 ng/ml phorbol 12‐myristate 13‐acetate for 48 h to differentiate into adherent macrophages. The cells were maintained in RPMI 1640 medium supplemented with 12% heat‐inactivated FBS, 100 U/ml penicillin and 100 mg/ml streptomycin and cultured in 5% CO_2_ at 37°C.

### 2.3 *Isolation* *of murine peritoneal macrophages (PM)*

Animal experiments were performed according to protocols approved by the Animal Care and Use Committee of Sichuan University, China. Female BALB/c mice (6–8 weeks old) were purchased from Dossy Corporation (Chengdu, China). The peritoneum‐derived macrophages were isolated from the peritoneal cavity of mice, as previously described.^25^ Briefly, the mice were killed and decapitated 72 h after receiving a 3 ml intraperitoneal injection of 3% thioglycollate. A small incision along the midline of the abdomen was immediately made, and the intact peritoneal wall was exposed. Five millilitres of the pre‐cooled serum‐free medium was injected into the abdominal cavity with a syringe and needle. Using the same syringe and needle, the lavage fluid from the lower peritoneal cavity was aspirated. Next, the lavage fluid was transferred into a pre‐cooled 50‐ml conical polypropylene centrifuge tube. This procedure was performed three to five times, and the peritoneal exudate cells were centrifuged at 1500 × g for 15 min. The cells were resuspended, counted and inoculated into the cell culture plates. The non‐adherent cells were washed away with phosphate buffered saline (PBS) after 12 h.

### 2.4 *RNA* *extraction and quantitative real*‐*time PCR (RT*‐*qPCR)*

Total RNA from cells was extracted using the UNIQ‐10 Trizol Total RNA Isolation Kit (Sangon Biotech), and its quality and quantity were measured with a spectrophotometer (NanoPhotometer, Implen). The cDNA was synthesized using the All‐in‐One cDNA Synthesis SuperMix kit, and amplification reactions were performed using the SYBR Green RT‐qPCR Master Mix on a fluorescence RT‐qPCR system (CFX96, Bio‐Rad). In this study, the *Actb* gene was chosen as the internal control, and the relative quantification of gene expression in each group was calculated using the 2^−△△CT^ method. The primer sequences used for RT‐qPCR are listed in Table 1.

**TABLE 1:** Primer sequences for RT‐qPCR.

Gene	Forward (5′−3′)	Reverse (5′−3′)
*Hmgn2*	CCATTGAAGAAGGGAGTTTGA	ATCAGAGGCAGCATTCCAAG
*Ulk1*	TGGAGGTGGCCGTCAAATG	CGCATAGTGTGCAGGTAGTC
*Actb*	GGCTGTATTCCCCTCCATCG	CCAGTTGGTAACAATGCCATGT

### 2.5 *Protein* *extraction and immunoprecipitation (IP) or Co*‐*immunoprecipitation (Co*‐*IP)*

The IP was used to detect HMGN2ac, while the Co‐IP was performed to observe the interaction between HMGN2 and H3K27ac. Briefly, the proteins were extracted from the cells by using the RIPA cell lysis buffer containing protease inhibitor (Biotool), phosphatase inhibitor (Biotool) and PMSF (Beyotime) and then centrifuged at 4°C and 12000× rpm for 15 min. The protein supernatants were incubated with primary antibodies overnight at 4°C and then co‐incubated with protein A/G agarose (Beyotime) for 4 h. The protein‐antibody‐beads complex was washed three times with RIPA cell lysis buffer, boiled and subjected to Western blotting analysis.

### 2.6 *Protein*‐*modified* *mass spectrometry*

After washing with PBS, the total protein was extracted from the cells. The acetylation sites of protein peptides were detected following reductive alkylation, proteolysis and protein extraction, desalting and freeze‐drying, liquid chromatography‐mass spectrometry (LC‐MS) assay, and data analysis (Trump, Inc., China).

### 2.7 *Plasmid* *construction and transfection*

According to the results of LC‐MS, the mouse‐derived PEX‐HMGN2 (WT HMGN2) plasmids and K2, K4, K53, K55 and K56 fully mutant PEX‐HMGN2 (5K‐R HMGN2) plasmids were provided by Genewiz. Primers, as shown in Table 2, were designed to mutate the K2 and K4 sites of the HMGN2 amino acid sequence to arginine (2K‐R) or to mutate the K53, K55 and K56 sites to arginine (3K‐R). The WT HMGN2 plasmids, together with the aforementioned primers, were added to the reaction system and amplified according to the manufacturer's instructions (TransGen Biotech). The PCR products were transformed into competent cells, and the 2K‐R HMGN2 and 3K‐R HMGN2 plasmids were obtained by sequencing.

**TABLE 2:** Sequences of primers for mutation of HMGN2.

	Forward (5′−3′)	Reverse (5′−3′)
2K‐R	ACCATGCCCAGAAGAAGGGCTGAAGGG	CCTTCTTCTGGGCATGGTGGCGAATTC
K53‐R	AAGGTACCCAGGGGGAGGAGG	CTGGGTACCTTCTCTCCCTTC
K55‐R	CCCAAGGGGAGGAGGGGGAAA	CCTCCCCTTGGGTACCTTCTC
K56‐R	AAGGGGAAGAGGGGGAAAGCT	CCTCTTCCCCTTGGGTACCTT

The plasmids with a final concentration of 1 μg/ml were then transfected into the KO RAW 264.7 cells using the jetPRIME kit (Polyplus‐transfection, France) according to the manufacturer's recommendations, and the culture medium was changed 6 h later. The transfection efficiency was verified 24 h post‐transfection by using a fluorescence microscope (Olympus FV‐10000).

### 2.8 *Western* *blotting (WB) analysis*

Proteins from cells were extracted using pre‐cooled RIPA lysis buffer (Beyotime), and the concentrations were measured using a bicinchoninic acid protein assay kit (KeyGen Biotech). Equal amounts of total proteins (20 μg) were separated by SDS‐PAGE and transferred onto a polyvinylidene fluoride membrane. After blocking with 5% bovine serum albumin (BSA) for 2 h at room temperature, the membranes were incubated with the appropriate concentrations of primary antibodies overnight at 4°C. After several washes, the membranes were incubated with the corresponding secondary antibodies for 2 h at room temperature and then visualized using enhanced chemiluminescence detection kit (Merck Millipore) on the ChemiDoc™ MP image system (Bio‐Rad).

### 2.9 *Immunofluorescence* *staining*

After washing with PBS, the cells were fixed with 4% paraformaldehyde for 20 min, followed by permeabilization with 0.5% Triton X‐100 for 15 min. Next, the cells were blocked with 5% BSA for 2 h at room temperature. Subsequently, cells were incubated with primary antibodies overnight at 4°C, followed by incubation with fluorescently labelled secondary antibodies at room temperature for 1 h in the dark. Nuclei were stained with DAPI. Finally, staining was visualized using a fluorescent microscope (Olympus FV‐10000) or a confocal microscope (Zeiss LSM710).

### 2.10 *Transmission* *electron microscopy*

The transmission electron microscopy (TEM) was used to directly observe autophagy‐specific structures. The cells were fixed in 2% glutaraldehyde in sodium cacodylate buffer overnight at 4°C and processed for TEM according to a previously described method.^26^

### 2.11 *Chromatin* *immunoprecipitation (ChIP) assay*

The chromatin immunoprecipitation analysis was carried out using a ChIP assay kit (Cell Signaling Technology) in accordance with the manufacturer's protocol. The cells were directly cross‐linked in the culture medium in 1% formaldehyde for 10 min at room temperature and then digested with 0.5 μl micrococcal nuclease. The obtained chromatin was disrupted into fragments of 150–900 bp by using an ultrasonic bioruptor (BILON 96‐IIL). Primary antibodies against HMGN2, H3K27ac and IgG were added to the corresponding ChIP samples. The PCR was performed using primers designed from the sequences of the mouse *Ulk1* promoter. The primer sequences were as follows: forward, 5′‐AGTCTCCGTCCCCACATACAG‐3′ and reverse 5′‐CTGGTCTCGAACTTGCTTTGTC‐3′. The data were normalized to the percentage of the input.

### 2.12 *Statistical* *analysis*

Data in this study were expressed as mean ± standard deviation (SD) and analysed using the GraphPad Prism 7 software. Values of *P* less than 0.05 were considered to be statistically significant, while n.s indicated no statistical difference.

## 3 RESULTS

### 3.1 *PYO* *induces autophagy in macrophages*

**FIGURE 1:**
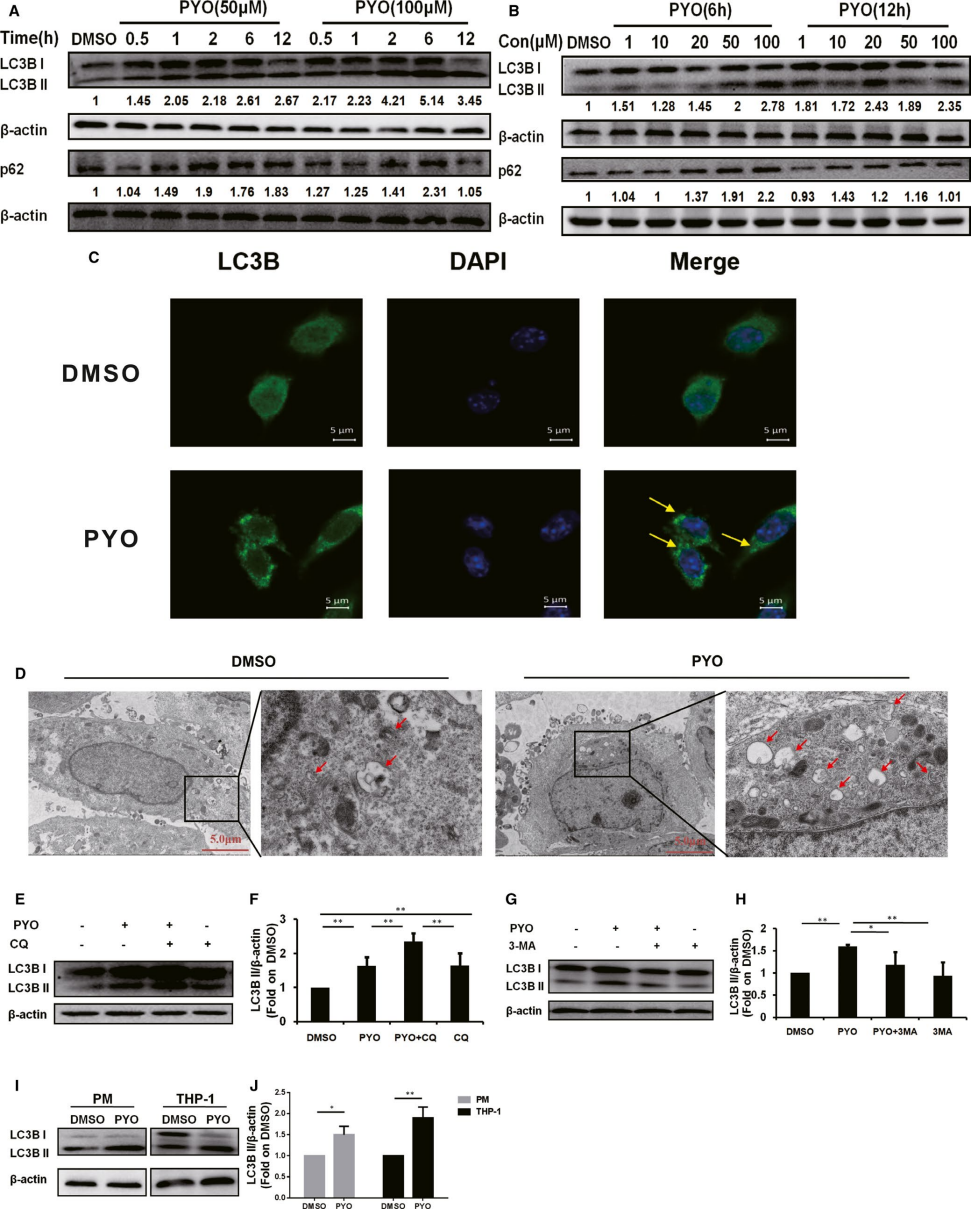
Pyocyanin induces autophagy in macrophages. (A) Western blot showing LC3B II and p62 protein in the RAW 264.7 cells treated with PYO (50 μM or 100 μM) at indicated time‐points or (B) treated with PYO (6 h or 12 h) at the indicated concentration. (C) Confocal microscopy images displaying the amount of intracellular LC3B puncta (green fluorescence, 630x) upon PYO (50 μM, 6 h) or DMSO treatment. The nucleus was stained using DAPI (blue fluorescence, 630x), scale bar = 5 μm. (D) The transmission electron microscopic images showing the formation of autophagosomes and autophagolysosomes upon PYO (50 μM, 6 h) or DMSO treatment. The right panel (6000x) is an enlarged image of the black box in the left panel (1500x), and arrows denote autophagosomes or autophagolysosomes, scale bar = 5 μm. (E) Western blot analysis showing LC3B II in RAW 264.7 cells upon PYO stimulation with or without CQ (1 μM), and (F) densitometric analysis showing relative expression normalized to the DMSO group. (G) Western blot analysis showing LC3B II in RAW 264.7 cells upon PYO stimulation with or without 3‐MA (2 mM), and (H) the densitometric analysis showing relative expression normalized by that of the DMSO group. (I) Western blot analysis showing LC3B II in PM and THP‐1 cells upon PYO (50 μM, 6 h) or DMSO treatment, and (J) the densitometric analysis showing relative expression normalized to the DMSO group. Data are expressed as mean ± SD, *P < 0.05 and **P < 0.01, n = 3, and DMSO is the control

To investigate whether PYO induced autophagy in RAW 264.7 cells, we first examined the protein levels of LC3B II and p62 in the RAW 264.7 cells in response to the different doses and time of PYO treatment (Figure 1A–B). As expected, PYO induced a significant increase in the expression of LC3B II and p62 in a dose‐ and time‐dependent manner; in particular, the increase in the expression of LC3B II was more significant after treatment with PYO at 50 µM for 6 h. Thus, the protocol with 50 µM PYO treatment for 6 h was chosen for subsequent experiments.

Consistent with immunoblotting results, our fluorescence microscopy and TEM data, respectively, displayed enhanced fluorescence densities of LC3B puncta and an increased number of autophagosomes in RAW 264.7 cells treated with PYO (Figure 1C–D). During autophagy, the increase in LC3B II protein level might be associated with the inhibition of lysosomes, which act at the end of the autophagic flux.^27^ Therefore, we simultaneously treated the RAW 264.7 cells with CQ (inhibitor of late‐stage autophagy flux) or 3‐MA (early‐stage autophagy flux inhibitor that inhibits PI3K) and PYO or DMSO and examined the protein level of LC3B II. We found that compared to the PYO group, the LC3B II protein level in the PYO +CQ group was significantly enhanced (Figure 1E–F), while that in the PYO +3‐MA group was significantly suppressed (Figure 1G–H). Collectively, these results indicated that PYO promoted autophagy flux in RAW 264.7 cells, rather than blocking the degradation of autolysosomes. To further determine whether PYO induced autophagy in macrophages from other sources, we treated the mouse‐derived primary peritoneal macrophages (PM) and THP‐1 human macrophages with PYO (Figure 1I–J). According to the immunoblotting data, PYO induced a significant increase in the expression of LC3B II in both PM and THP‐1 cells, suggesting that PYO also induced autophagy in macrophages from other sources.

### 3.2 *PYO* *up*‐*regulates HMGN2ac in the macrophages*

**FIGURE 2:**
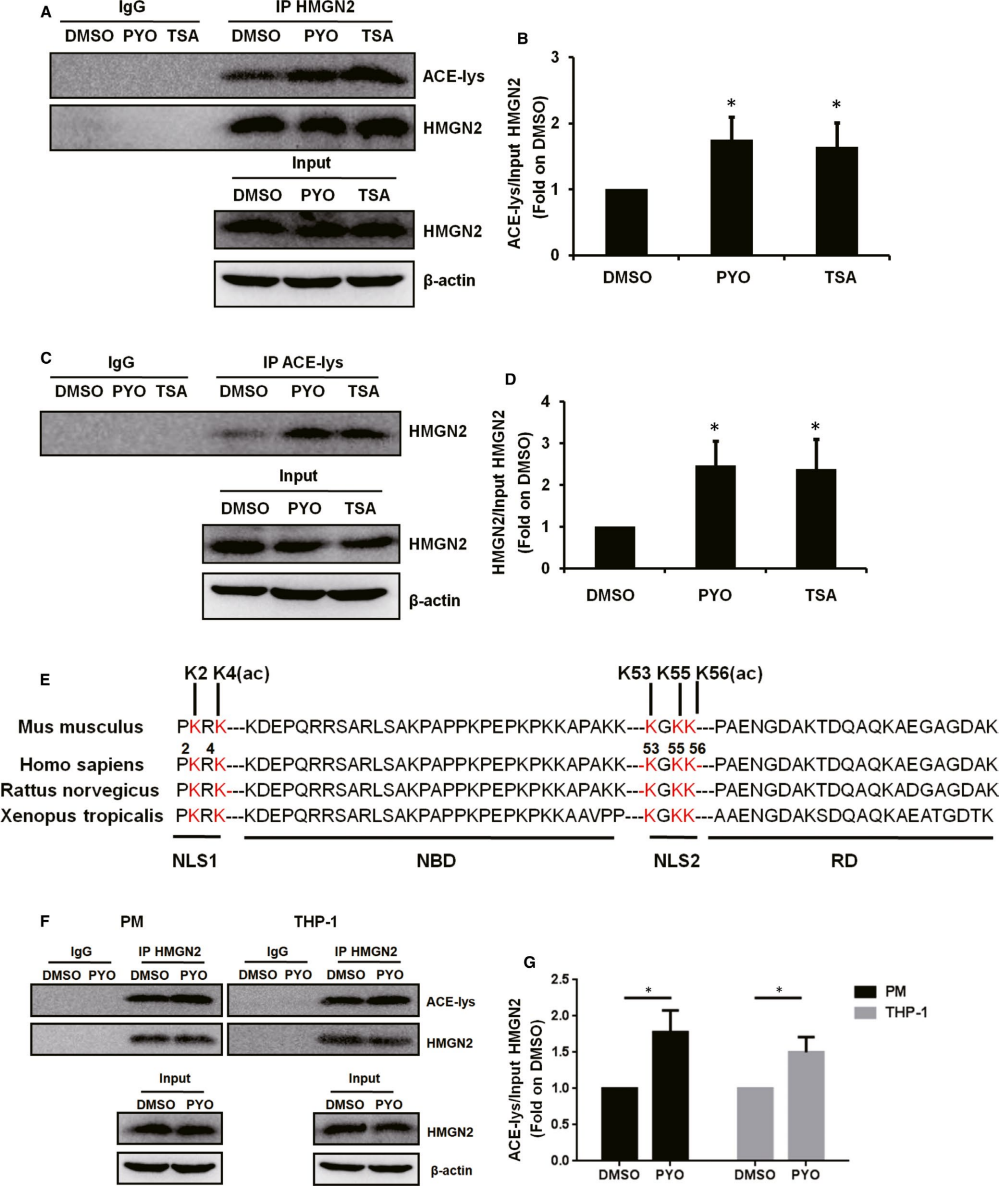
PYO up‐regulates HMGN2ac in macrophages. RAW 264.7 cells were treated with 50 μM PYO or DMSO for 6 h, and TSA (50 nM) was used as a positive control for HMGN2ac.^49^ (A) IP showing the acetylation of lysine (ACE‐lysine) with (B) densitometric analysis showing the relative expression normalized by that of the DMSO group. (C) IP showing the HMGN2 with (D) densitometric analysis showing the relative expression normalized by the DMSO group. (E) The LC/MS mass spectrometer displaying the acetylation modification sites of HMGN2, and the amino acid sequences of HMGN2 of different species were compared and the acetylation sites were marked. NLS: nuclear localization signal domain, NBD: nucleosome binding domain and RD: regulatory domain. PM and THP‐1 cells were treated both with 50 μM PYO and with DMSO for 6 h, (F) IP HMGN2 showing the ACE‐lysine with (G) the densitometric analysis showing relative expression normalized by DMSO group. Data are expressed as mean ± SD, *P < 0.05 compared with the DMSO group, n = 3

We then applied an IP assay to determine whether PYO induced the acetylation modification of HMGN2. After enrichment with anti‐HMGN2 antibody, the proteins were subjected to WB assay for the detection of pan‐acetylation levels (Figure 2A–B), and then, the anti‐lysine acetylation antibody was used to enrich the acetylated proteins (Figure 2C–D). Finally, WB was performed to detect HMGN2. The results showed that PYO up‐regulated HMGN2ac in the RAW 264.7 cells. To further identify the acetylation site of HMGN2, we performed an LC‐MS assay. As indicated in Figures S1A and S1B, PYO induced lysine acetylation in two peptide sequences of HMGN2, namely MPKRK and VPKGKK. Next, we compared these peptide sequences to the mouse‐derived HMGN2 amino acid sequences (UniProt number: P09602) and found that the lysine acetylation sites were K2, K4, K53, K55 and K56 (Figure 2E), all of which are located in the nuclear localization signal (NLS).^28^ In addition, we performed an identical IP assay on the PM and THP‐1 cells, and the results showed that PYO up‐regulated HMGN2ac in both types of macrophages (Figure 2F–G). We planned to further explore the regulatory roles of these lysine acetylation sites on autophagy in subsequent experiments.

### 3.3 *Influence* *of HMGN2ac on PYO*‐*induced autophagy in RAW 264*.*7 cells*

**FIGURE 3:**
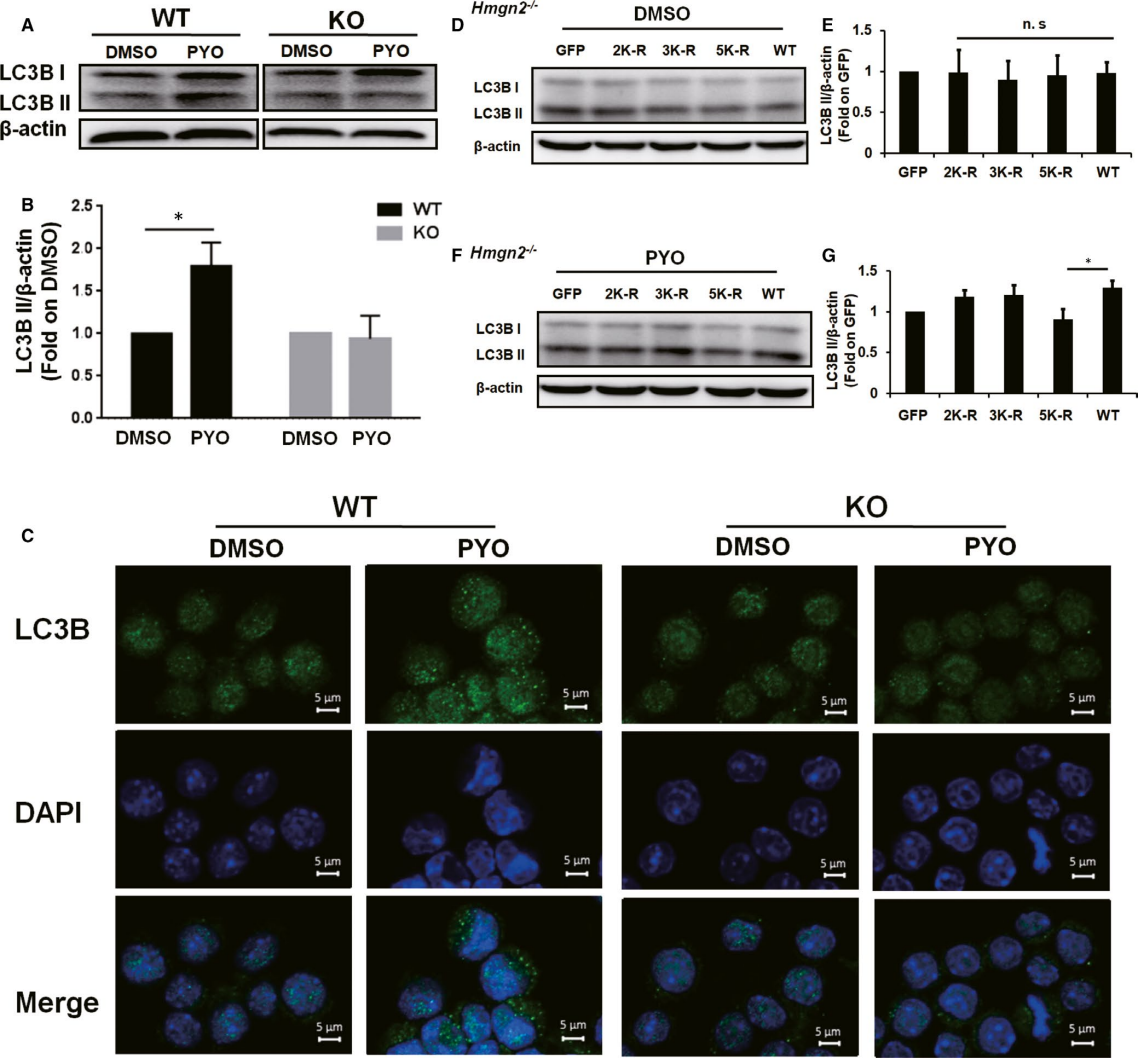
Effect of HMGN2ac on the PYO‐mediated autophagy in the RAW 264.7 cells. The WT and KO RAW 264.7 cells were treated with 50 μM PYO or DMSO for 6 h. (A) Western blot showing LC3B II protein in the RAW 264.7 cells with or without *Hmgn2* ^−/−^ upon PYO (50 μM, 6 h) or DMSO treatment, and (B) densitometric analysis showing relative expression normalized to that of the DMSO group. (C) Confocal microscopy images displaying the amount of intracellular LC3B puncta (green fluorescence, 630x) with or without *Hmgn2* ^−/−^ upon PYO (50 μM, 6 h) or DMSO treatment. The nucleus was stained by DAPI (blue fluorescence, 630x), scale bar = 5 μm. (D, F) KO RAW 264.7 cells were transfected, respectively, with the GFP, 2K‐R, 3K‐R, 5K‐R and WT HMGN2 plasmids using jetPRIME for 24 h and then incubated both with 50 μM PYO and with DMSO for 6 h. The Western blot analysis showing the LC3B II protein. (E, G) Densitometric analysis showing relative expression normalized to that of the GFP plasmid. Data are expressed as mean ± SD, *P < 0.05, and n.s indicates no statistical difference, n = 3

Herein, we first performed WB assay to analyse the LC3B II protein level in KO RAW 264.7 cells and wild‐type (WT) RAW 264.7 cells which were exposed to PYO. Although the protein level of LC3B II was significantly increased by PYO in WT RAW 264.7 cells, the protein level of LC3B II was not affected by PYO in the KO RAW 264.7 cells (Figure 3A–B). Consistently, the fluorescence densities of LC3B puncta were significantly enhanced by PYO in WT RAW 264.7 cells; however, in KO RAW 264.7 cells, the fluorescence densities of LC3B puncta were not affected (Figure 3C). Thus, our results indicate that PYO cannot induce autophagy in the *Hmgn2* ^−/−^ RAW 264.7 cells.

Next, to further demonstrate the role of HMGN2 quantity and HMGN2ac in PYO‐induced autophagy, we stimulated the KO RAW 264.7 cells that transfected with green fluorescent protein (GFP) plasmid, HMGN2 mutant (2K‐R, 3K‐R, 5K‐R) plasmid or WT HMGN2 plasmid with PYO or DMSO and examined the protein level of LC3B II. The transfection efficiency in our experiments was approximately 50% (Fig. S2A–B). Our results showed that there was no significant difference in the protein level of LC3B II in each tested group upon DMSO treatment (Figure 3D–E), suggesting that HMGN2 quantity does not affect autophagy when there is no acetylation of HMGN2. In contrast, after treatment with PYO, the protein level of LC3B II was significantly lower in the 5K‐R group than in the WT group (Figure 3F–G), indicating that simultaneous acetylation of these five sites in the HMGN2 NLS region might be related to autophagy caused by PYO.

### 3.4 *PYO* *down*‐*regulates H3K27ac in macrophages*

**FIGURE 4:**
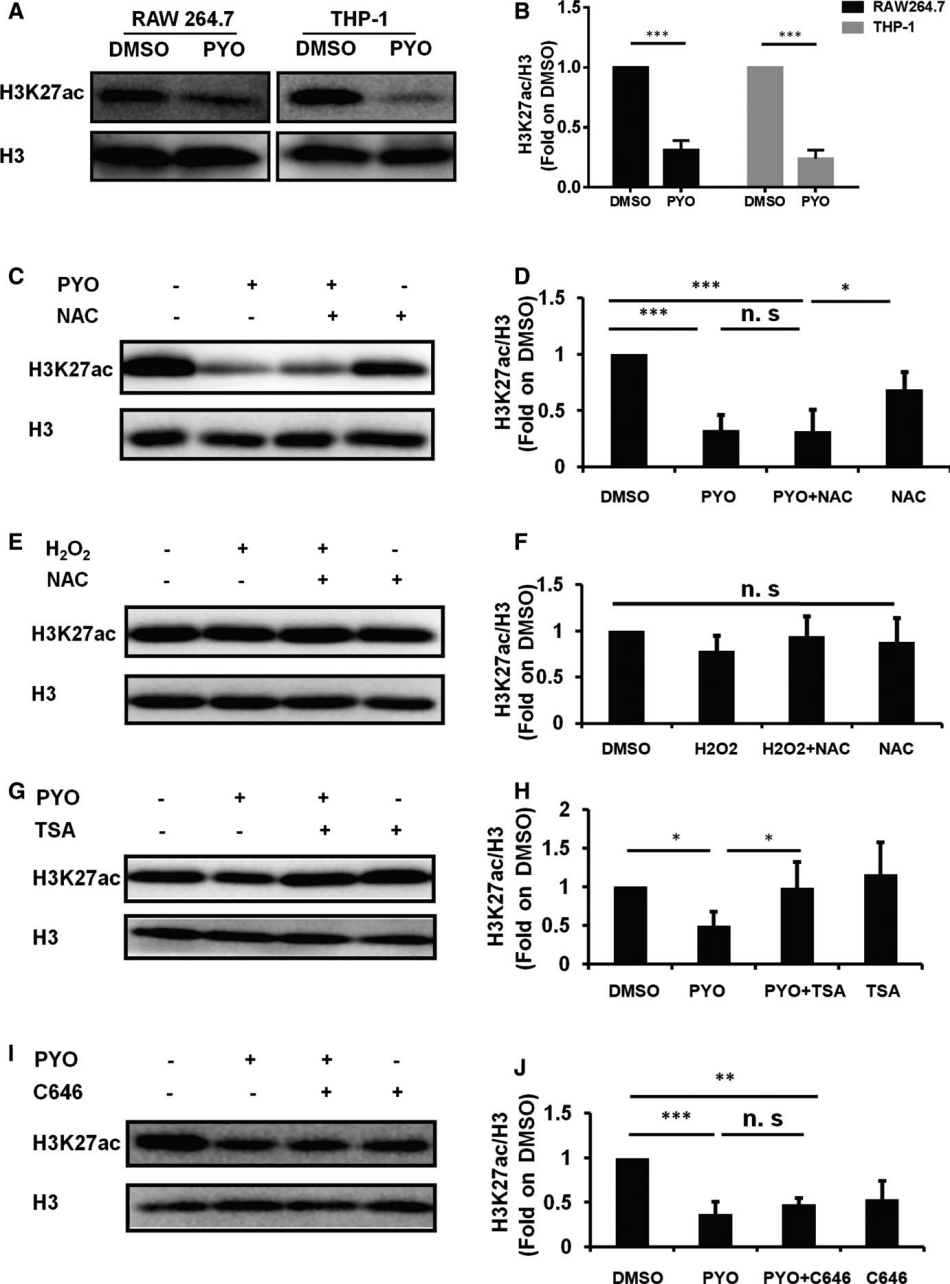
PYO down‐regulates H3K27ac in macrophages. The RAW 264.7 cells were treated, respectively, with 50 μM PYO and DMSO for 6 h. (A) Western blot showing H3K27ac in the RAW 264.7 cells and THP‐1 cells treated both with PYO and with DMSO. (C) Western blot analysis showing the H3K27ac protein level in RAW 264.7 cells both with and without NAC (inhibitor of ROS, 50 μM) upon PYO stimulation. (E) Western blot analysis showing H3K27ac protein level in the RAW 264.7 cells both with and without NAC (50 μM) upon H_2_O_2_ stimulation. (G) Western blot analysis showing the H3K27ac protein level in the RAW 264.7 cells both with and without TSA (20 nM) upon PYO stimulation. (I) Western blot analysis showing H3K27ac protein level in the RAW 264.7 cells both with and without C646 (10 μM) upon PYO stimulation. (B, D, F, H, J) Densitometric analysis showing relative expression normalized by that of the DMSO group. Data are expressed as mean ± SD, *P < 0.05, **P < 0.01 and ***P < 0.001, and n.s indicates no statistical difference, n = 3

Previous studies have demonstrated that PA induces acetylation modification of histone,^29^ and the knockout of the *Hmgn2* gene affected the expression of H3K27ac,^30^ which participates in the regulation of transcription of autophagy‐related genes.^31^ We, therefore, conducted a WB assay to examine the H3K27ac protein level in the RAW 264.7 cells and THP‐1 cells that were exposed to PYO. Exposure of the RAW 264.7 cells and THP‐1 cells to PYO caused a significant reduction in H3K27ac levels compared to the exposure to DMSO (Figure 4A–B). Thus, our data suggest that PYO down‐regulates H3K27ac in macrophages.

Both PYO and H_2_O_2_ are factors inducing ROS, and NAC is a classic ROS inhibitor.^32^ Moreover, our previous series of studies have demonstrated that PYO induces the generation of ROS in the pulmonary epithelial cells, RAW 264.7 cells and primary cultured pulmonary macrophages.^22^, ^33^ Therefore, we sought to determine whether the down‐regulation of H3K27ac was attributable to the generation of ROS. Our results showed that there was no significant difference in the H3K27ac levels between the PYO +NAC and PYO groups (Figure 4C–D) or between the H_2_O_2_ and DMSO groups (Figure 4E–F). Taken together, our results demonstrated that the down‐regulation of H3K27ac induced by PYO in RAW 264.7 cells was not associated with ROS generation.

In addition, TSA (an inhibitor of histone deacetylase) and C646 (inhibitor of HAT p300) were introduced to investigate the relationship between the activities of acetyltransferases/deacetylases and the down‐regulation of H3K27ac. The results showed that the H3K27ac protein level in the PYO +TSA group was twice that in the PYO group (Figure 4G–H), while no significant difference was observed between the PYO +C646 and PYO groups (Figure 4I–J). Our results suggest that PYO‐induced down‐regulation of H3K27ac in RAW 264.7 cells might be related to the up‐regulation of histone deacetylase.

### 3.5 *HMGN2ac* *does not interact with H3K27ac*

**FIGURE 5:**
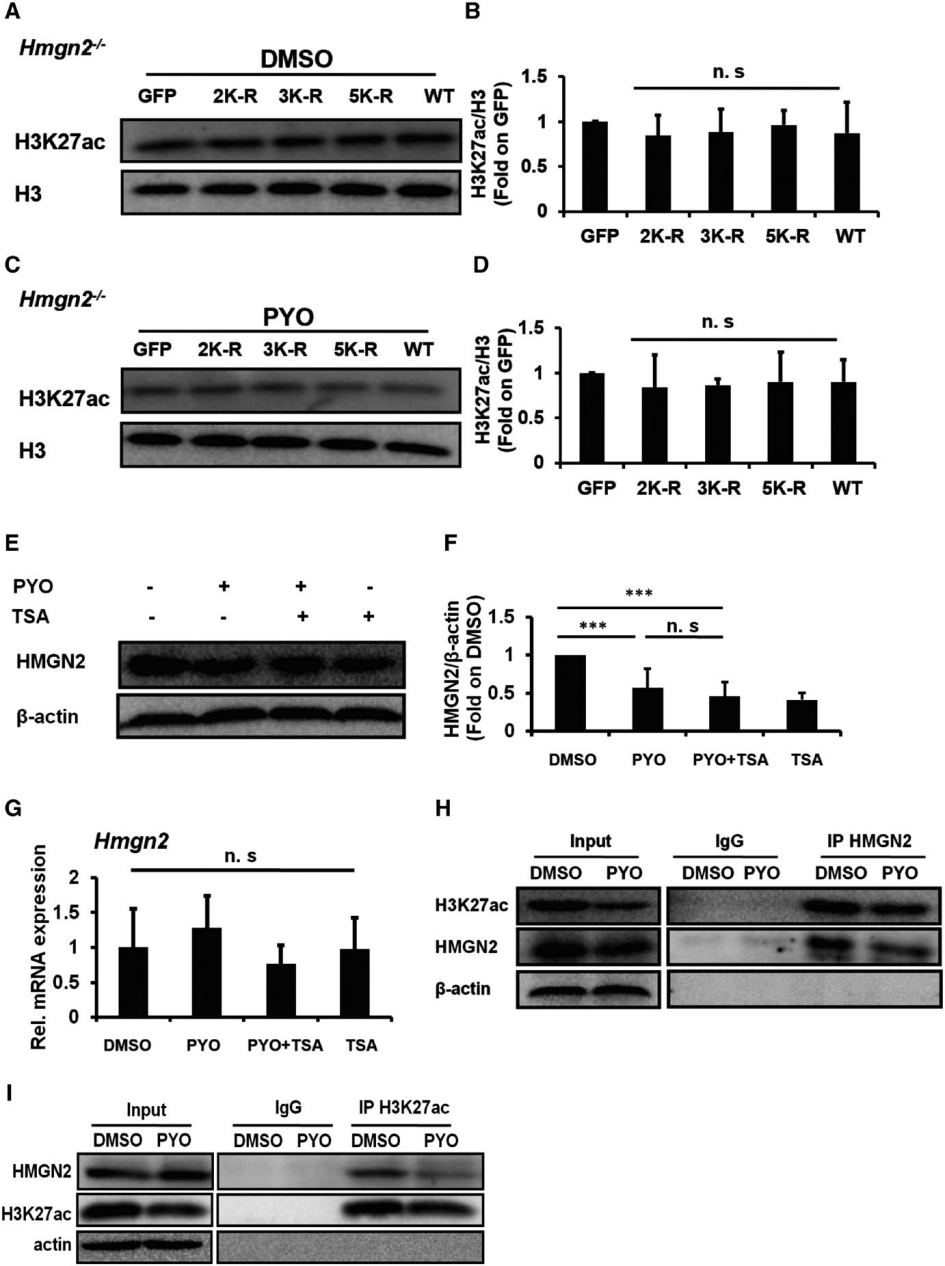
HMGN2ac might have no interaction with H3K27ac. The KO RAW 264.7 cells were transfected, respectively, with GFP, 2K‐R, 3K‐R, 5K‐R and WT HMGN2 plasmids by using jetPRIME, for 24 h, and then incubated with both 50 μM PYO and DMSO for 6 h. The Western blot showing H3K27ac in RAW 264.7 cells treated both with DMSO (A) and with PYO (C), and (B, D) the densitometric analysis showing relative expression normalized by the GFP plasmid. (E) Western blot showing HMGN2 protein in the RAW 264.7 cells both with and without TSA (20 nM) upon PYO stimulation, and (F) densitometric analysis showing relative expression normalized to that of the DMSO group. (G) RT‐qPCR displaying the *Hmgn2* mRNA in the RAW 264.7 cells treated with PYO (50 μM, 6 h), and DMSO is the control. (H‐I) Co‐IP showing the interaction of HMGN2 with H3K27ac. Data are expressed as mean ± SD, ***P < 0.001, and n.s indicates no statistical difference, n = 3

To test the involvement of HMGN2ac in the regulation of H3K27ac expression, we examined the H3K27ac by transfecting KO RAW 264.7 cells, respectively, with the GFP plasmid, HMGN2 mutant plasmid and WT HMGN2 plasmid and then stimulating the KO RAW 264.7 cells both with PYO and with DMSO. To our surprise, there were no significant differences in the H3K27ac in each tested group both under the stimulation of DMSO (Figure 5A–B) and under the stimulation of PYO (Figure 5C–D), suggesting that HMGN2ac might have no regulatory effect on H3K27ac.

Since TSA was found to restore the down‐regulation of H3K27ac caused by PYO (Figure 4G–H), we speculated that if the expression of HMGN2 was increased by TSA, then H3K27ac may have a regulatory effect on HMGN2. However, neither the protein nor the mRNA levels of HMGN2 were significantly changed in the PYO +TSA group compared to that in the PYO group (Figure 5E–G), suggesting that H3K27ac may have no regulatory effect on HMGN2. In addition, our Co‐IP data showed a reduction in the interaction of HMGN2 with H3K27ac after PYO treatment (Figure 5H–I), suggesting that HMGN2 and H3K27ac do not interact to a certain extent. Thus, our results indicate that HMGN2ac and H3K27ac might independently participate in the regulation of autophagy.

### 3.6 HMGN2ac and H3K27ac modulate autophagy by regulating the transcription of the *Ulk1* gene

**FIGURE 6:**
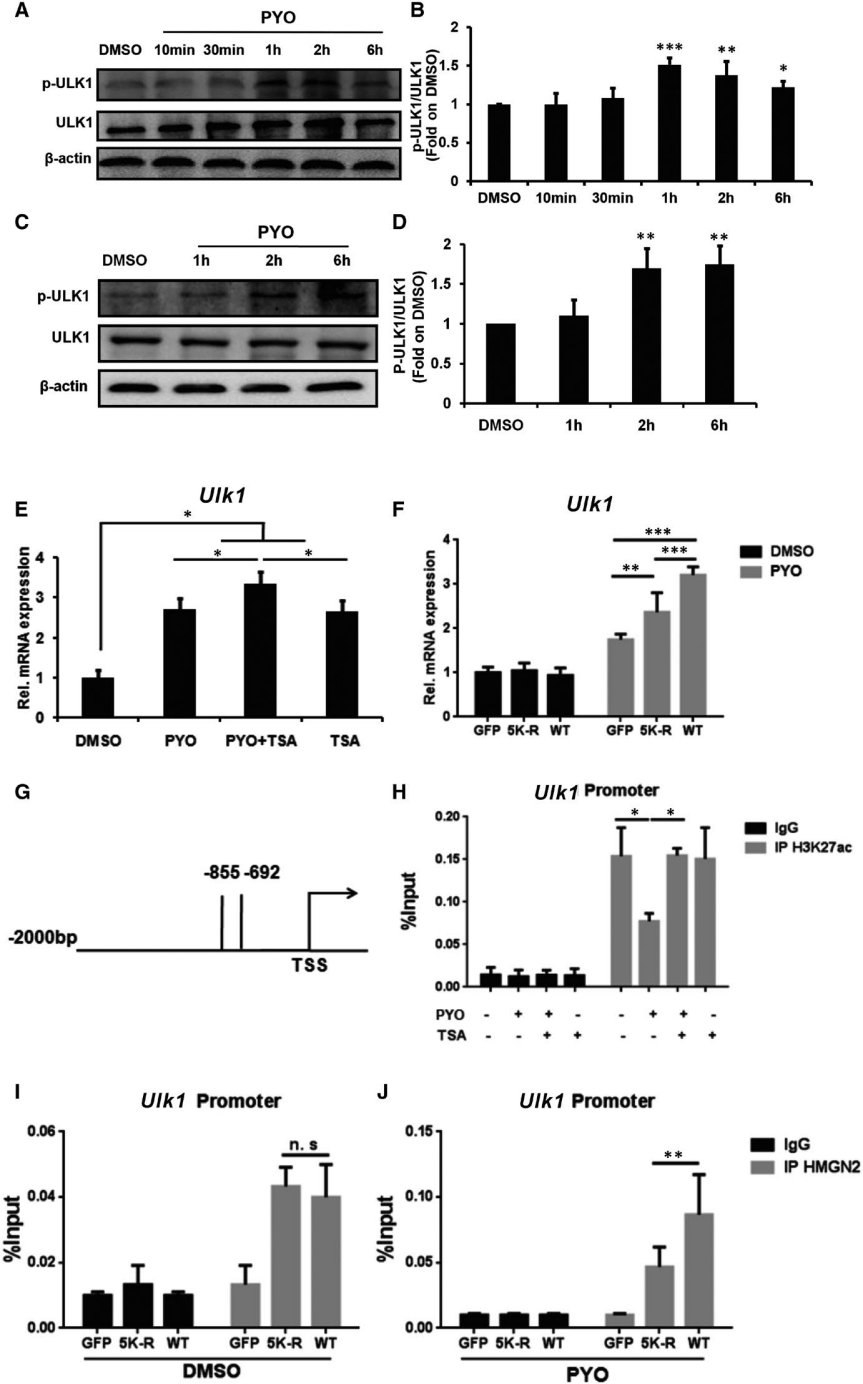
HMGN2ac and H3K27ac regulate *Ulk1* transcription during autophagy. Western blot showing p‐ULK1 and ULK1 in RAW 264.7 cells (A) and PM cells (C) treated with 50 μM PYO at indicated time‐points, and (B, D) densitometric analysis showing relative expression normalized by the DMSO group. (E) RT‐qPCR analysis showing the relative *Ulk1* mRNA level in the RAW 264.7 cells treated both with and without TSA (20 nM) upon PYO (50 μM, 6 h) stimulation. (F) The KO RAW 264.7 cells were transfected, respectively, with GFP, 5K‐R and WT HMGN2 plasmids by using jetPRIME for 24 h and then incubated both with 50 μM PYO and with DMSO for 6 h. RT‐qPCR showing the relative *Ulk1* mRNA. (G) The schematic diagram depicting primer amplification sites in ChIP‐qPCR. TSS, transcription start site; −855 to −692, area of promoter region for primer designing. (H) ChIP‐qPCR showing the H3K27ac recruitment at the *Ulk1* promoter in the RAW 264.7 cells treated both with and without TSA (20 nM) upon PYO (50 μM, 6 h) stimulation. (I, J) The KO RAW 264.7 cells were transfected respectively with GFP, 5K‐R, and WT HMGN2 plasmids by using jetPRIME for 24 h, then the cells were treated both with 50 μM PYO and with DMSO for 6 h. The ChIP‐qPCR analysis showing the HMGN2ac recruitment at the *Ulk1* gene promoter. Data are expressed as mean ±SD, *P < 0.05, **P < 0.01 and ***P < 0.001, and n.s indicates no statistical difference, n = 3

ULK1 is a key protein for the initiation of autophagy, which has been demonstrated to participate in the modulation of HMGN2 in the bladder epithelial cells^23^, ^34^ Therefore, we chose ULK1 for the subsequent study. To confirm the involvement of ULK1 in PYO‐induced autophagy, we analysed the protein level of p‐ULK1 (S555) in the RAW 264.7 cells exposed to PYO at different time‐points. The results showed that the expression of p‐ULK1 peaked at 1 h and then gradually declined from 2 h to 6 h, but was still higher at 6 h than that in the DMSO group (Figure 6A–B). Consistently, the p‐ULK1 protein levels in the PM cells were significantly increased upon PYO treatment for 2 h and 6 h (Figure 6C–D). Taken together, our results suggest that PYO induces autophagy in macrophages via ULK1.

Since TSA was found to restore the down‐regulation of H3K27ac in RAW 264.7 cells, caused by PYO (seen in Figure 4G–H), we next simultaneously treated the RAW 264.7 cells with TSA and PYO or DMSO and examined the mRNA expression of *Ulk1*. Our results showed that, compared to the PYO group, the increase in *Ulk1* mRNA level in the PYO +TSA group was significantly enhanced (Figure 6E), indicating that H3K27ac may have a regulatory effect on the transcription of *Ulk1*. To assess the role of HMGN2 in the regulation of *Ulk1* transcription, we stimulated the KO RAW 264.7 cells that were transfected with the GFP plasmid, 5K‐R mutant plasmids or WT HMGN2 plasmid with PYO or DMSO and examined the mRNA level of *Ulk1*. As shown in Figure 6F, although the mRNA level of *Ulk1* was not affected by DMSO in each transfected group, the mRNA level of *Ulk1* was significantly increased under the stimulation of PYO in the WT group compared to the 5K‐R group. Thus, our results suggest that the mutation of five lysine sites in HMGN2 to arginine affects the transcription of the *Ulk1* gene.

We then conducted a ChIP‐qPCR assay in the promoter region of *Ulk1* (Figure 6G) to determine whether H3K27ac regulates PYO‐induced autophagy by affecting the transcription of *Ulk1*. The results showed that PYO led to a reduction in H3K27ac recruitment at the *Ulk1* promoter in the RAW 264.7 cells (Figure 6H), thereby inhibiting the transcription of *Ulk1*. Next, we sought to determine whether HMGN2ac regulates the transcription of *Ulk1*. According to our ChIP‐qPCR data, although there was no obvious change in HMGN2 recruitment at the *Ulk1* promoter in each transfected group under the stimulation of DMSO (Figure 6I), PYO resulted in enhanced HMGN2 recruitment at the *Ulk1* promoter in the WT group compared to that in the 5K‐R group, thus promoting the transcription of *Ulk1* (Figure 6J). Collectively, our results showed that PYO regulated the transcription of *Ulk1* by down‐regulating H3K27ac and up‐regulating HMGN2ac.

## 4 DISCUSSION

Oxidative stress and inflammation have been reported as core events during PA infection.^32^, ^35^, ^36^ Our previous study has shown that HMGN2 attenuates PYO‐induced oxidative stress via the Nrf2 signalling cascade and inhibits the internalization of PA in the lung epithelial cells.^22^ In addition, our previous data from the study on bladder epithelial cell line 5637 infected with *Escherichia coli* also indicated that HMGN2 induces autophagy by activating the AMPK/ULK1 pathway, which facilitates the proliferation of *Escherichia coli*.^23^ Macrophages are one of the most important innate immune cells. Autophagy induced by pathogen infection is known to be involved in a series of biological events, including macrophage activation and polarization, apoptosis, mitochondrial metabolism, inflammasome assembly, the release of inflammatory cytokines, bacterial clearance and autophagic death. Therefore, in the present study, we established a PYO‐induced autophagy model in macrophages to explore the role and mechanism of HMGN2ac and H3K27ac during autophagy.

In this study, we verified that PYO can induce autophagy in macrophages from several aspects, including detecting the autophagy markers with WB assay and observing the formation of autophagosomes by using the confocal microscopy and TEM, as well as the introduction of autophagy flux inhibitors, such as CQ (inhibits the fusion of autophagosomes and lysosomes) and 3‐MA (inhibits the formation of autophagosomes). During autophagy, p62 delivers ubiquitinated proteins or bacteria to autophagosomes for degradation and is subsequently degraded in the autolysosome.^37^ Of note, the cellular level of p62 is regulated by various biological processes. For instance, oxidative stress can up‐regulate the cellular p62 levels through multiple signalling pathways, such as Nrf2, Ras/MAPK and JNK/c‐Jun.^38^ Moreover, PYO was found to promote Nrf2 nuclear translocation as well as oxidative stress in the RAW 264.7 cells and lung epithelial cells.^22^, ^33^ Therefore, PYO‐induced oxidative stress may account for the up‐regulation of p62 observed in this study, which resulted in more activated p62 than that were autophagic degraded. A growing amount of evidence indicates that macrophages can eliminate bacteria via autophagy, while the absence of p62 can improve the survival of bacteria, which is supposed to be the biological meaning of the elevated p62 levels observed in this study.^39^, ^40^

PA has been shown to induce autophagy in the epithelial cells and macrophages.^8^, ^41^ As for PYO infection, however, current studies have only focused on the neurocytes and epithelial cells. Specifically, in bronchial epithelial cells, exogenous PYO‐induced autophagy relies on the EIF2AK4‐EIF2S1‐ATF4 pathway.^11^ PYO‐induced autophagy has also been observed in astrocytoma and neuroblastoma cells. Although many signalling molecules have been identified to be associated with macrophage autophagy, their exact role in the autophagic process remains unclear.

It has been verified that the P300/CBP‐associated factor (PCAF) and p300 mediate the acetylation modification of HMGN2.^42^, ^43^ The acetylation of HMGN2 reduces its binding capacity to nucleosomes and affects epigenetic modifications.^43^ In neural stem cells, knocking out the *Hmgn2* gene results in a global reduction in H3K9ac and disrupts the profiles of H3K4me3, H3K9ac, H3K27ac and H3K122ac at the *Nanog* and *Oct4* loci. Nevertheless, in the endodermal/mesodermal genes, the *Hmgn2* ^−/−^ cells exhibit a switch from a bivalent to a repressive chromatin configuration.^30^ Collectively, these studies suggest that HMGN2 and its acetylation modification can regulate both chromatin structures and histone acetylation, which might affect its interaction with certain transcription factors at the promoter or enhancer regions.

Although the LC‐MS data revealed that the acetylation of the five lysine sites located in the HMGN2 NLS region is necessary for PYO to induce autophagy in macrophages, we did not further examine the HMGN2 conformational changes as well as the impact of these conformational changes on the PYO‐induced autophagy. Thus, we speculate that the acetylation of the five lysine sites in the HMGN2 NLS region caused by PYO may at least partly affect the binding of HMGN2 to nucleosomes or DNA, thus regulating autophagy. Nonetheless, the roles of conformational changes in HMGN2 in this process cannot be theoretically excluded.

It has been well documented that PA mediates acetylation of histone H3 and H4, resulting in lung injury.^44^, ^45^ H3K27ac is involved in the regulation of autophagy, and its up‐regulation is a sign of gene transcription activation. It has been reported that the oncoprotein of the Epstein‐Barr virus recruits histone activation epigenetic markers, including H3K27ac, to affect the transcription of autophagy‐related genes.^31^ Moreover, in plants, the deacetylation of H3K27 was found to be correlated with the inhibition of autophagy.^46^ Based on previous *in vitro* biochemical studies, some scholars have noted that HMGN2 acetylation occurs before histone acetylation.^43^ In this study, we discovered that PYO simultaneously up‐regulates HMGN2ac and down‐regulates H3K27ac. Unfortunately, our data showed that there was no interaction between HMGN2ac and H3K27ac and that they may independently participate in the regulation of autophagy. However, the above conclusion is based only on indirect evidence, and the exact relationship between HMGN2ac and H3K27ac needs to be further studied.

ULK1/ATG1, among the first of over 30 autophagy‐related proteins identified,^47^ is the key promoter of autophagy.^34^ The stress signal kinase AMPK and protein kinase mTORC1 were found to positively and negatively regulate *Ulk1*, respectively, participating in the modulation of cellular responses to a wide range of stimuli, such as nutrition and starvation.^48^ Our previous experiments confirmed that HMGN2 regulates autophagy in the bladder epithelial cells through the AMPK/ULK1 pathway.^23^ In this study, our data showed that PYO regulates the transcription of *Ulk1* by down‐regulating H3K27ac and up‐regulating HMGN2ac, which indicates an important role of HMGN2ac and H3K27ac in the maintenance of homeostasis of *Ulk1* transcription.

In conclusion, we found that PYO induced autophagy in macrophages by up‐regulating HMGN2ac and down‐regulating H3K27ac. We further demonstrated that the up‐regulated HMGN2ac promoted its recruitment at the *Ulk1* promoter region, which ultimately enhanced the transcription of *Ulk1*, while the down‐regulation of H3K27ac resulted in a decrease in its recruitment at the *Ulk1* promoter region, thereby suppressing the transcription of *Ulk1*. Overall, HMGN2ac and H3K27ac can regulate PYO‐induced macrophage autophagy.

## CONFLICT OF INTEREST

The authors declare no conflicts of interest.

## AUTHOR CONTRIBUTIONS

**Yu Du:** Data curation (equal); Investigation (equal); Methodology (equal); Project administration (equal); Writing‐original draft (lead). **Hong jun Guo:** Data curation (equal); Investigation (equal); Methodology (equal); Project administration (equal). **Li juan Guo:** Investigation (supporting); Methodology (supporting); Project administration (supporting). **Jun ming Miao:** Investigation (supporting); Methodology (supporting); Project administration (supporting). **Hongyu Ren:** Investigation (supporting); Methodology (supporting). **Ke yun Liu:** Formal analysis (supporting); Funding acquisition (supporting); Resources (supporting). **Lai bin Ren:** Investigation (supporting); Methodology (supporting); Project administration (supporting). **Jin chen He:** Investigation (supporting); Project administration (supporting). **Xiaoying Wang:** Conceptualization (supporting); Resources (supporting); Visualization (lead). **Jun li Chen:** Conceptualization (supporting); Data curation (supporting); Formal analysis (equal); Software (lead). **Jing yu Li:** Conceptualization (supporting); Data curation (supporting); Supervision (supporting). **Yi Wang:** Conceptualization (equal); Project administration (supporting); Validation (equal). **Ji Wang:** Formal analysis (equal); Funding acquisition (supporting); Project administration (equal); Resources (equal); Supervision (equal); Writing‐review & editing (equal). **Ning Huang:** Conceptualization (lead); Funding acquisition (lead); Resources (equal); Supervision (equal); Validation (equal); Writing‐review & editing (equal).

## Supporting information

Fig S1

Fig S2
