# Supplementary material for: The regulatory effect of acetylation of HMGN2 and H3K27 on pyocyanin‐induced autophagy in macrophages by affecting Ulk1 transcription
Source: J Cell Mol Med. 2021 Jul 18;25(15):7524–37. doi: 10.1111/jcmm.16788 (PMC8335688; doi:10.1111/jcmm.16788)
Supplement: Supplementary file 1 — Fig S1 [file JCMM-25-7524-s002.pdf]

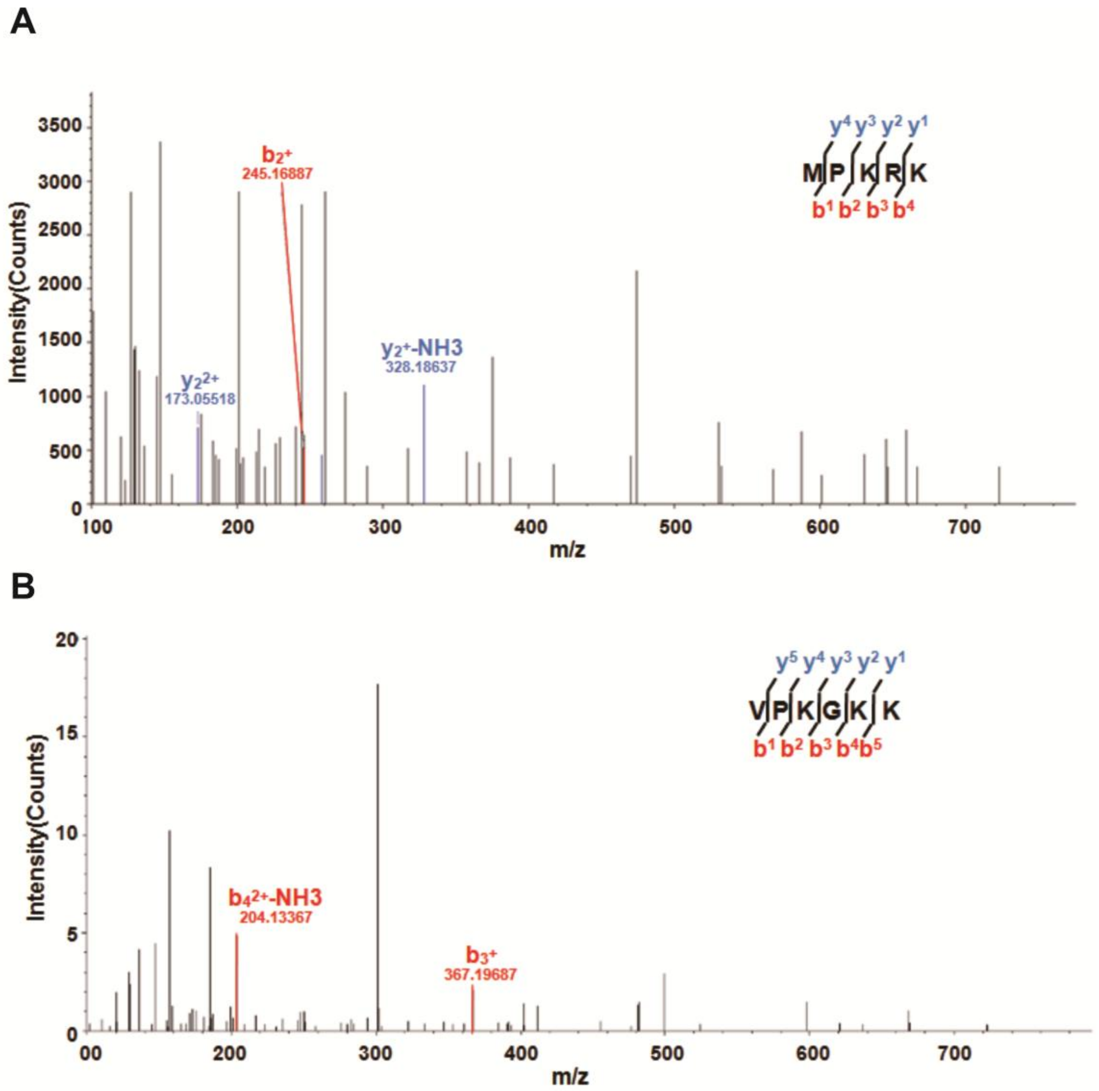

Figure S1. Mass spectrum identification of the acetylated lysine in HMGN2 peptide. (A) Mass analysis of the MPKRK peptide sequence of HMGN2 following lysine acetylation at K2 and K4. (B) Mass analysis of the VPKGKK peptide sequence of HMGN2 following lysine acetylation at K53, K55, and K56. b and y represent the position and direction of the peptide fragment, respectively; + represents the positive charge. The x-axis represents the mass-to-charge ratio (m/z), and the y-axis represents the abundance (Count).
