# Supplementary material for: The regulatory effect of acetylation of HMGN2 and H3K27 on pyocyanin‐induced autophagy in macrophages by affecting Ulk1 transcription
Source: J Cell Mol Med. 2021 Jul 18;25(15):7524–37. doi: 10.1111/jcmm.16788 (PMC8335688; doi:10.1111/jcmm.16788)
Supplement: Supplementary file 2 — Fig S2 [file JCMM-25-7524-s001.pdf]

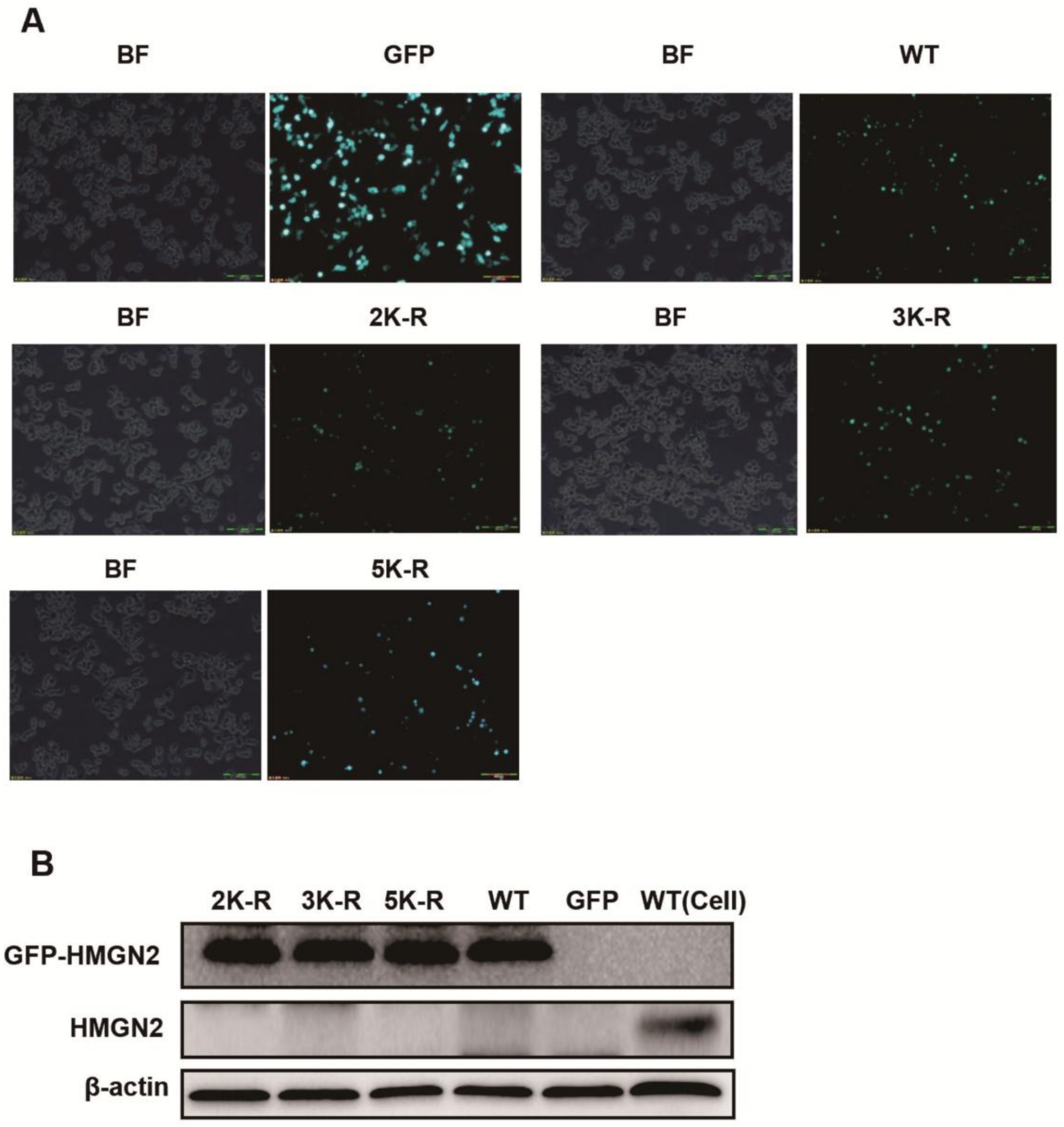

Figure S2. The transfection efficiency was verified by immunofluorescence and Western blotting. The KO RAW 264.7 cells were transfected respectively with GFP, 2K-R, 3K-R, 5K-R, and WT HMGN2 plasmids for 24 h. (A) The microscopic images displaying transfection efficiency of plasmids in each group (100x), scale bar = 200 μm. (B) Western blot showing the HMGN2 protein level in each group, and WT RAW 264.7 cells were used as a positive control.
